# High throughput single-cell genome sequencing gives insights into the generation and evolution of mosaic aneuploidy in *Leishmania donovani*

**DOI:** 10.1093/nar/gkab1203

**Published:** 2021-12-10

**Authors:** Gabriel H Negreira, Pieter Monsieurs, Hideo Imamura, Ilse Maes, Nada Kuk, Akila Yagoubat, Frederik Van den Broeck, Yvon Sterkers, Jean-Claude Dujardin, Malgorzata A Domagalska

**Affiliations:** Molecular Parasitology Unit, Institute of Tropical Medicine, Antwerp, Belgium; Molecular Parasitology Unit, Institute of Tropical Medicine, Antwerp, Belgium; Molecular Parasitology Unit, Institute of Tropical Medicine, Antwerp, Belgium; Molecular Parasitology Unit, Institute of Tropical Medicine, Antwerp, Belgium; MiVEGEC, University of Montpellier, CNRS, IRD, Montpellier, France; MiVEGEC, University of Montpellier, CNRS, IRD, Montpellier, France; Molecular Parasitology Unit, Institute of Tropical Medicine, Antwerp, Belgium; Department of Microbiology, Immunology and Transplantation, Rega Institute for Medical Research, Katholieke Universiteit Leuven, 3000 Leuven, Belgium; MiVEGEC, University of Montpellier, CNRS, IRD, Montpellier, France; Molecular Parasitology Unit, Institute of Tropical Medicine, Antwerp, Belgium; Department of Biomedical Sciences, University of Antwerp, Belgium; Molecular Parasitology Unit, Institute of Tropical Medicine, Antwerp, Belgium

## Abstract

*Leishmania*, a unicellular eukaryotic parasite, is a unique model for aneuploidy and cellular heterogeneity, along with their potential role in adaptation to environmental stresses. Somy variation within clonal populations was previously explored in a small subset of chromosomes using fluorescence hybridization methods. This phenomenon, termed mosaic aneuploidy (MA), might have important evolutionary and functional implications but remains under-explored due to technological limitations. Here, we applied and validated a high throughput single-cell genome sequencing method to study for the first time the extent and dynamics of whole karyotype heterogeneity in two clonal populations of *Leishmania* promastigotes representing different stages of MA evolution *in vitro*. We found that drastic changes in karyotypes quickly emerge in a population stemming from an almost euploid founder cell. This possibly involves polyploidization/hybridization at an early stage of population expansion, followed by assorted ploidy reduction. During further stages of expansion, MA increases by moderate and gradual karyotypic alterations, affecting a defined subset of chromosomes. Our data provide the first complete characterization of MA in *Leishmania* and pave the way for further functional studies.

## Graphical Abstract

**Graphical Abstract:**
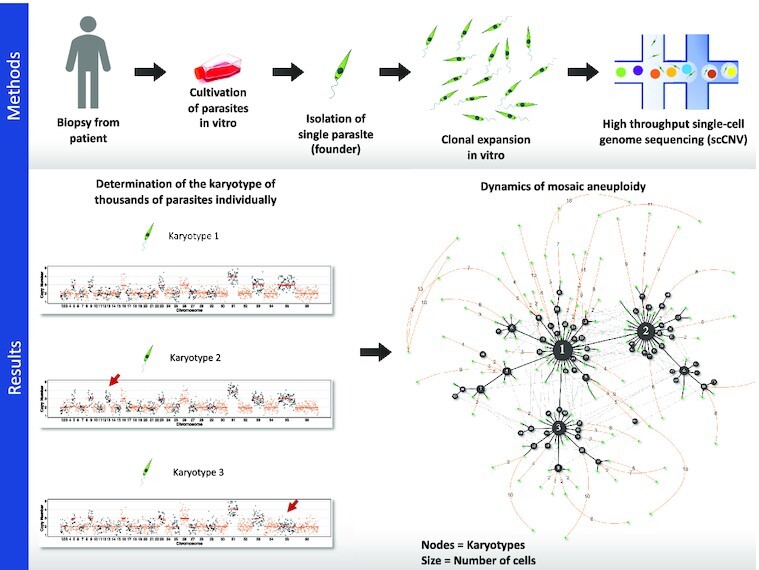
Overview of the methodological approach (top) and the main results (bottom).

## INTRODUCTION

Aneuploidy, i.e. an imbalance in the copy number of chromosomes in a cell, occurs in a wide range of organisms, including both non- and pathogenic unicellular eukaryotes, such as *Saccharomyces cerevisiae, Candida albicans, Cryptococcus neoformans* and *Leishmania* spp., but also in different types of human cancer cells (1–6). Although generally considered to be detrimental in multicellular organisms, aneuploidy can also be beneficial, in particular for unicellular organisms facing drastic changes in the environment (7,8). In pathogens, aneuploidy facilitates rapid adaptation to environmental stresses through changes in gene dosage and may have an impact on both virulence and the development of drug resistance (7,9–14).

*Leishmania*, a genus of digenetic protozoan parasites, is emerging as a unique model for aneuploidy (15). These parasites are responsible for a spectrum of clinical forms of leishmaniasis worldwide and cause 300,000 new cases per year (16). They can be found in two forms during their life cycle: as an extracellular promastigote in the midgut of phlebotomine sand fly vectors and exclusively as intracellular amastigote inside mammalian host phagocytic cells. Thus, *Leishmania* parasites are adapted to these two drastically different environments. From a molecular point of view, *Leishmania*, as other trypanosomatids, is unique in the Eukaryota domain (17). This includes the genomic organization in long polycistronic units, the near absence of transcription initiation regulation by RNA polymerase II promoters, and a remarkable genomic plasticity (14,18). The *Leishmania* genome is generally considered to be diploid, although all *Leishmania* genomes analyzed hitherto display aneuploidy affecting at least one chromosome, i.e., a polysomy in chr31. Moreover, high levels of ‘average’ aneuploidy (average will be used throughout this paper for features derived from bulk analyses of population of cells) affecting other chromosomes are commonly found by bulk genome sequencing of *in vitro* cultured promastigotes of all *Leishmania* species tested so far (1,2). This average aneuploidy is highly dynamic and changes when cultivated parasite populations are exposed to different environments such as the vector, the vertebrate host or in response to drug pressure (19–21). In fact, changes in average aneuploidy pattern and not variation in nucleotide sequence are the first genomic modifications observed at populational level during the course of experimental selection of drug resistance (21,22). Given that these alterations in average somies are reflected in the average amount of corresponding transcripts (19,23), and to a certain degree, of proteins (24), it has been proposed that aneuploidy allows *Leishmania* to adapt by means of rapid changes in gene dosage.

*Leishmania* parasites exhibit a remarkable cellular heterogeneity in the form of mosaic aneuploidy, where individual daughter cells originating from a single parent (i.e., a clonal population) may display distinct somies (3,25). The full extent of mosaic aneuploidy in *Leishmania* and its dynamics during adaptation to new environment remains largely unexplored due to technological limitations. The only estimation of karyotype heterogeneity was based on the FISH studies of a small set of chromosomes, where it was speculated that thousands of karyotypes may co-exist in a clonal population of *Leishmania* promastigotes (3). Mosaicism was proposed to provide a source of functional diversity within a population of *Leishmania* cells, through gene dosage, but also through changes in heterozygosity (23,26). This diversity of karyotypes would provide an adaptive potential to unpredictable environmental changes during the parasite's life cycle or drug pressure caused by patient treatment (23,26).

FISH-based pioneer studies should be complemented and refined by single cell approaches of whole genome sequencing. In a previous study, we made a first step in that direction by combining FACS-based sorting of single *Leishmania* cells with whole genome amplification (WGA) and whole genomic sequencing (WGS). In that pilot study, we evaluated different WGA and bioinformatic methods and were able to successfully call somy of all chromosomes in 28 single *Leishmania* cells (27). Here, we applied and validated for the first time a high throughput, droplet-based platform for single cell genome sequencing (SCGS) of thousands of individual *Leishmania* promastigotes. This allowed a first assessment of the degree and the dynamics of the evolution of mosaic aneuploidy in two clonal populations *in vitro* representing different stages of expansion in culture conditions. Based on our study, we propose that the early stages of expansion are characterized by rapid and drastic changes in karyotypes, allowing initial establishment of highly aneuploid cells in a population of almost euploid parasites. In the next steps, the existing highly aneuploid karyotypes further evolve through gradual and moderate changes in somies resulting in a population of aneuploid cells displaying closely related karyotypes. Our findings strongly support the hypothesis that mosaic aneuploidy is a constitutive feature of *Leishmania* parasites, representing a unique source of functional diversity.

## MATERIALS AND METHODS

### Parasites

In the present paper, we use the terms population, strain and clone, following the nomenclature of salivarian trypanosomes (28). Accordingly: (i) a population is a group of *Leishmania* cells present at a given time in a given culture or host; (ii) a strain is a population derived by serial passage in vitro from a primary isolate (in our case, from patient samples) without any implication of homogeneity but with some degree of characterization (in our case bulk genome sequencing); (iii) a clone is derived from a strain and is a population of cells derived from a single individual presumably by binary fission. *L. donovani* promastigotes were maintained at 26°C in HOMEM medium (Gibco, ThermoFisher) supplemented with 20% Fetal Bovine Serum, with regular passages done every 7 days at 1/25 dilutions. The clones BPK282 cl4 and BPK081 cl8 were derived from two strains adapted to culture: MHOM/NP/02/BPK282/0 and MHOM/NP/02/BPK081/0 (29). These clones were submitted to SCGS at 21 (∼126 generations) and 7 passages (∼56 generations) after cloning respectively (Supplementary Figure S1). Four strains were mixed to create an artificial ‘super-mosaic’ population of cells (further called super-mosaic): BPK475 (MHOM/NP/09/BPK475/9), BPK498 (MHOM/NP/09/BPK498/0), BPK506 (MHOM/NP/09/BPK506/0) and HU3 (MHOM/ET/67/HU3). They were kept *in vitro* for several passages after isolation from patients (respectively 41, 60, 47 and >24) and mixed at equivalent ratio just before preparation for SCGS.

### Single-cell suspensions preparation and sequencing

Promastigotes at early stationary phase (day 5) were harvested by centrifugation at 1000 rcf for 5 min, washed twice with PBS 1× (calcium and magnesium-free) + 0.04% BSA, diluted to 5 × 10^6^ parasites/mL and passed through a 5 μm strainer to remove clumps of cells. After straining, volume was adjusted with PBS 1× + 0.04% BSA to achieve a final concentration of 3 × 10^6^ parasites/ml. The absence of remaining cell doublets or clumps in the cell suspension was confirmed by microscopy. Cell viability was estimated by flow cytometry (BD FACSverse™) using the NucRed™ Dead 647 probe (Life technologies™) following the recommendations of the manufacturer and in all samples was estimated as higher than 95%. SCGS was performed using the Chromium™ single-cell CNV solution (scCNV) from 10X Genomics™. To target an average of 2000 sequenced cells per sample, 4.2 μl of the cell suspensions were used as input, and cell encapsulation, barcoding, whole genome amplification and library preparation were performed following manufacturer's recommendations. Sequencing of the libraries was done with an Illumina NovaSeq™ SP platform with 2 × 150 bp reads.

### Single-cell somy estimation

Details about the bioinformatic analysis for somy values determination are provided in the supplementary material. In summary, sequence reads were associated to each sequenced cell based on their barcodes and mapped to a customized version of the reference *L. donovani* genome LdBPKv2 (19) using the Cell Ranger DNA^TM^ software (10× Genomics). The matrix generated by the software with the number of mapped reads per 20 kb bins was used as input to a custom script written in R (30). In this script, bins with outlier values were excluded, and the mean normalized read depth of each chromosome was calculated for each cell. Cells displaying a high intra-chromosomal variation were removed from downstream analysis. In order to establish the baseline ploidy of each cell and define the somies of a cell, the mean normalized depth values of the cell's chromosomes were multiplied by the cell scale factor, defined for each cell as the lowest number between 1.8 and 5 that leads to the shortest distances to integers when the chromosomes’ mean normalized depth values are multiplied by it. The scaled mean normalized depth values are referred here as ‘raw somies’. To convert the raw somies (continuous) into integer copy numbers (discrete), a univariate gaussian mixture-model was built for each chromosome by an expectation-maximization algorithm based on the distribution of the raw somy values between all cells of the same sample using the Mixtools package (31). For each possible integer somy, a gaussian mixture-model was generated and each raw somy value was assigned to the rounded mean of the gaussian to which it has higher probability of belonging to.

### Karyotype identification and network analysis

A karyotype was defined as the combination of integer somies of all chromosomes in a cell. Karyotypes were numerically named according to their frequency in the sequenced population. To generate the network representing the dissimilarities between the karyotypes, a pairwise distance matrix was built based on the number of different chromosomes between all karyotypes in a sample, and used to create a randomized minimum spanning tree with 100 randomizations, using the Pegas R package (32,33). The network visualization was made with the visNetwork package (34).

### Doublet detection

The relative fraction of doublets within the super mosaic population has been estimated based on the high number of single nucleotide polymorphisms (SNPs) found in the HU3 strain when compared to the *L. donovani* reference genome. The three other strains in the super mosaic only show a limited number of SNPs in contrast. Potential doublets were identified by looking for mixture of both SNP profiles (HU3 and non-HU3) in assumed single-cell data. This approach was applied using an in-house developed algorithm and the Demuxlet algorithm (35), both approaches leading to identical results (see Supplementary Text).

### DNA probes and fluorescence in situ hybridization

DNA probes were either cosmid (L549 specific of chromosome 1) or BAC (LB00822 and LB00273 for chromosomes 5 and 22, respectively) clones that were kindly provided by prof. Peter Myler (Center for Infectious Disease Research, formerly Seattle Biomedical Research Institute) and Christiane Hertz‐Fowler (Sanger Centre). DNA was prepared using Qiagen Large‐Construct Kit and labelled with tetramethyl‐rhodamine‐5‐dUTP (Roche Applied Sciences) by using the Nick Translation Mix (Roche Applied Sciences) according to manufacturer instructions. *Leishmania* cells were fixed in 4% paraformaldehyde then air‐dried on microscope immunofluorescence slides, dehydrated in serial ethanol baths (50–100%) and incubated in NP40 0.1% for 5 min at RT. Around 100 ng of labelled DNA probe was diluted in hybridization solution containing 50% formamide, 10% dextran sulfate, 2 × SSPE, 250 μg.mL^−1^ salmon sperm DNA. Slides were hybridized with a heat‐denatured DNA probe under a sealed rubber frame at 94°C for 2 min and then overnight at 37°C and sequentially washed in 50% formamide/2× SSC at 37°C for 30 min, 2× SSC at 50°C for 10 min, 2× SSC at 60°C for 10 min, 4× SSC at room temperature. Finally, slides were mounted in Vectashield (Vector Laboratories) with DAPI. Fluorescence was visualized using appropriate filters on a Zeiss Axioplan 2 microscope with a 100× objective. Digital images were captured using a Photometrics CoolSnap CCD camera (Roper Scientific) and processed with MetaView (Universal Imaging). Z‐Stack image acquisitions (15 planes of 0.25 μm) were systematically performed for each cell analyzed using a Piezo controller, allowing to view the nucleus in all planes and to count the total number of labelled chromosomes. Around 200 cells [187–228] were analyzed per chromosome.

### Bulk genome sequencing

Genomic DNA from the BPK282 cl4 and BPK081 cl8 clones was extracted in bulk using the QIAmp™ DNA Mini kit (Qiagen) following manufacturer's recommendations. PCR-free whole genome sequencing was performed on the Illumina NovaSeq platform using 2 × 150 bp paired reads. Reads were mapped to the reference genome *L. donovani* LdBPKv2 (available at ftp://ftp.sanger.ac.uk/pub/project/pathogens/Leishmania/donovani/LdBPKPAC2016beta/) using BWA (version 0.7.17) with seed length set to 100 (36). Only properly paired reads with a mapping quality higher than 30 were selected using SAMtools (37). Duplicates reads were removed using the RemoveDuplicates command in the Picard software (http://broadinstitute.github.io/picard/). The average somy values were calculated as described previously (1), by dividing the median sequencing depth of a chromosome by the overall median sequencing depth over all chromosomes, and multiplying this ratio by 2. These values were used to define an average karyotype for the sequenced population of cells.

### Gene Ontology analysis and in silico screening for small RNA

Gene Ontology (GO) classes were obtained from TriTrypDB release 49 (38). As the genome sequence stored on TriTrypDB does not correspond to the reference genome used in this work, the GO annotation was obtained by mapping back all genes to our reference genome using BlastP (39). Clustering of the different chromosomes based on their assigned GO classes was performed using the prcomp command in R.

Two different data sources were used for non-coding RNA screening. First, the Rfam (40) database version 14.4 was used to screen the *L. donovani* LdBPKv2 reference genome using the cmscan algorithm as implemented in Infernal (41) using default parameters and setting the search space parameter to 64. Second, the non-coding RNA nucleotide sequences as annotated in the *L. donovani* BPK282A1 genome on TriTrypDB version 54 were extracted, and aligned versus the reference genome used in this work (*L. donovani* LdBPKv2). Next, both data sources were compared with each other using BLAST, and redundant copies were removed (i.e., only one copy retained to avoid duplicates).

## RESULTS

### High throughput single-cell genome sequencing as a reliable tool to explore karyotype heterogeneity in *Leishmania* populations

We applied high throughput single-cell genome sequencing (SCGS) to address mosaic aneuploidy in promastigotes of two *Leishmania* clones differing substantially in average aneuploidy as revealed by bulk genome sequencing: (i) BPK282 cl4, an aneuploid clone showing seven chromosomes with an average trisomy apart from the usual average tetrasomy in chr31 and (ii) BPK081 cl8, showing an average disomy for all chromosomes except chr31 (average tetrasomic); for simplicity, we will call BPK081 cl8 the ‘diploid’ clone. Details about sequencing statistics are provided in Supplementary Table S1. First analyses of the SCGS data were made with the Cell Ranger DNA™ pipeline. Although the software was developed for mammalian genomes, which are up to 2 orders of magnitude larger than *Leishmania*’s nuclear genome, it allowed detecting (i) aneuploidy, (ii) mosaicism and (iii) large intrachromosomal CNVs, as, for instance, the H- and M- amplicons (1) in chr23 and chr36 respectively (Supplementary Figure S2). However, technical artifacts were noticed especially in BPK081 cl8, where the software's GC bias correction algorithm, designed for the mammalian genome which display a lower average GC (41%) content compared to *Leishmania* (60%), ended up overcompensating the depth of bins with high GC content (Supplementary Figure S2). Because of that and given our main goal of using SCGS to study mosaic aneuploidy, we built our own analytical bioinformatic pipeline with a higher emphasis on estimating whole chromosomes copy numbers rather than local CNVs (Supplementary Figure S3).

**Figure 1.**
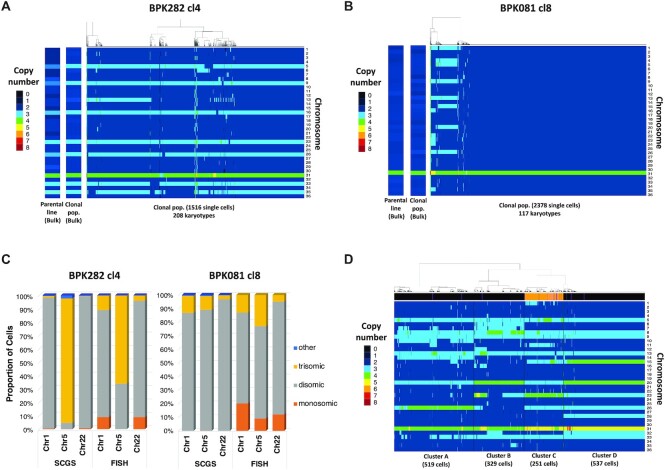
Mosaic aneuploidy in BPK282 cl4 and BPK081 cl8 clones revealed by SCGS and validation of the method. (**A, B**) Heat maps displaying the copy number of all 36 chromosomes of promastigotes from BPK282 cl4 (A) or BPK081 cl8 (B) clones (main panels). Each column represents a single parasite. The number of sequenced promastigotes and karyotypes found in each sample is described in the x axis. In each panel, two insets display the average aneuploidy profile (bulk) of the clonal population used in the SCGS and their respective parental strain. (**C**) Comparison between FISH and SCGS. The proportion of cells displaying monosomy, disomy or trisomy for chromosomes 1, 5 and 22 in each method is represented. Around 200 cells [187–228] were analyzed per chromosome in FISH. (**D**) Heat map displaying the karyotypes of the promastigotes from four different strains mixed in a single SCGS run. Cells were hierarchically clustered according to their karyotypes, forming 4 major clusters. The number of cells in each cluster is indicated in the x axis. The bar at the top of the heatmap indicate if the SNP profile of the cell corresponds to a BPK strain (black), a HU3 strain (orange) or a doublet (purple).

We evaluated the SCGS method and our analytical pipeline by first addressing their ability to explore karyotype heterogeneity among *Leishmania* cells of clones BPK282 cl4 and BPK081 cl8. Using our analytical pipeline, we identified 208 different karyotypes among the 1516 filtered cells of BPK282 cl4 and 117 karyotypes among the 2378 filtered cells of BPK081 cl8 (Figure 1A, B, Supplementary Figure S4A, B). Moreover, the cumulative SCGS profile of each clone was consistent with their respective average aneuploidy profile (Figure 1A and B, left panel). Notably, chr13, which displays a non-integer average somy value (2.26) in BPK282 cl4, was found as disomic and trisomic at relatively high proportions in the SCGS, resulting in a similar cumulative somy (2.34). As expected, the vast majority of cells in BPK081 cl8 displayed an almost diploid karyotype, with only chr31 showing a tetrasomy as expected. Small subpopulations of cells displaying highly aneuploid karyotypes were also observed in BPK081 cl8 (discussed below).

Mosaic aneuploidy in *Leishmania* has been studied so far with fluorescence in situ hybridization (FISH), the only alternative method available hitherto to estimate the copy number of some chromosomes in individual *Leishmania* cells. As a mutual benchmark of both FISH and SCGS methods, we submitted cells from both BPK282 cl4 and BPK081 cl8 to FISH to estimate the copy number of chromosomes chr1, chr5 and chr22 and to compare the obtained results with the values observed in our SCGS data (Figure 1C). Overall, for each chromosome, the same predominant somy was observed with both methods, even when the predominant somy was different between clones. For instance, FISH and SCGS report chr5 in BPK282 cl4 as trisomic in most cells, while it is reported as mainly disomic in BPK081 cl8 also by both techniques. Most discrepancies between the proportions obtained by both methods are within the 10% error margin previously estimated for FISH (3 and unpublished results). The main exception is chr5 in BPK282 cl4, which is estimated as trisomic in 93% of the cells with SCGS and 66% with FISH. However, SCGS reports proportions which are more consistent with the average somy values observed in the bulk analysis of each clone. For instance, the weighted mean between somy values obtained with SCGS for chr5 in BPK282 cl4 results in an average somy of 2.95, which is very similar to the average somy value observed in bulk (2.97), whereas with FISH, the average somy is lower (2.66), suggesting that the proportions observed with SCGS are more accurate.

In order to test whether SCGS did not underestimate mosaicism, because of potential biases in the amplification of some chromosomes, we chose 4 strains characterized by a high average aneuploidy—previously assessed by bulk genome sequencing (29)—and mixed them together to create a ‘super-mosaic’ population which was submitted to a single SCGS run. A total of 1900 promastigotes were individually sequenced, of which, 1636 remained after data filtering. This ‘super mosaic’ population displayed a high aneuploidy diversity: 388 identified karyotypes in total. As expected, the 1636 promastigotes formed four distinct clusters based on their integer somy values, with discrete differences in the aneuploidy patterns between each cluster (Figure 1D). Since one of the strains (HU3) used in this super mosaic is phylogenetically distant from the other three strains (BPK475, BPK498 and BPK506), we could distinguish HU3 promastigotes from the others based on their SNP profiles. Interestingly, all HU3 cells were grouped together in cluster C (Figure 1D—orange lines in the annotation bar), suggesting that the discrete karyotypic differences between the major clusters reflect differences among the aneuploidy profiles of the four strains, so that each cluster likely represents one of the strains. Thus, this experiment demonstrates that SCGS is effective in distinguishing karyotypes even in very complex populations.

### BPK282 cl4 and BPK081 cl8 cells display different patterns of karyotype evolution during clonal expansion

**Figure 2.**
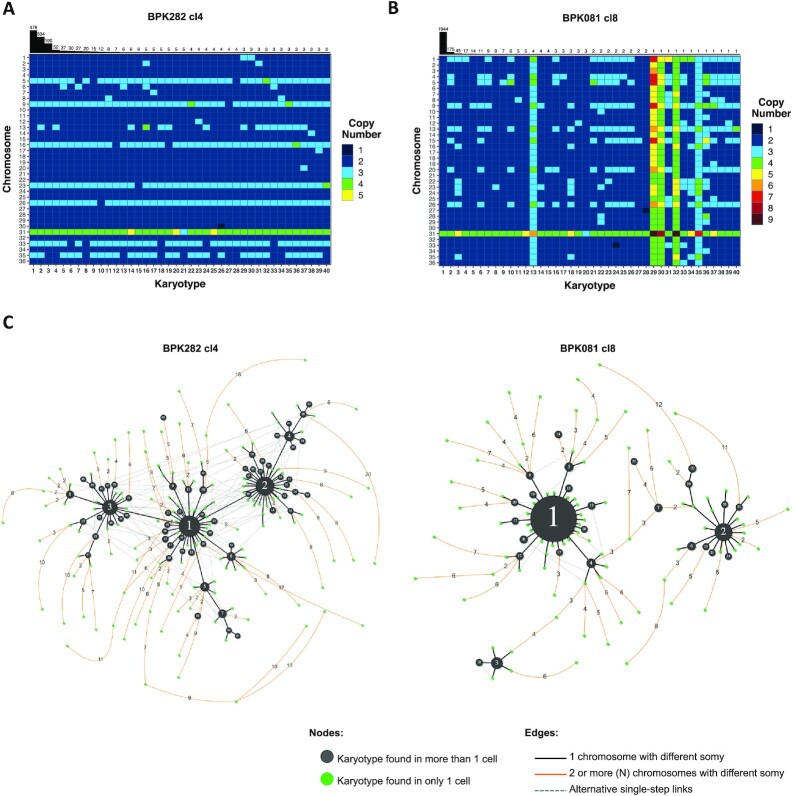
BPK282 cl4 and BPK081 cl8 display different profiles in the dissimilarity relationship between karyotypes. (**A, B**) Heat map depicting the 40 most frequent karyotypes in BPK282 cl4 (A) and BPK081 cl8 (B) clones. The bars at the top indicate how many cells are found with each depicted karyotype. (**C**) Network representing the dissimilarity relationship between karyotypes in each clone. Black nodes represent karyotypes found in more than one cell, with their size proportional to the number of cells. Green nodes indicate karyotypes which occur only once. Black lines link two karyotypes which diverge by a somy difference in a single chromosome, while orange lines link karyotypes diverging by two or more chromosomes with different somy, with the number of divergent chromosomes indicated in the edge. Dashed grey lines show alternative links between karyotypes with a single somy divergency. Polyploid karyotypes were not included in the networks.

After validating the SCGS method for resolving complex karyotype heterogeneity in *Leishmania*, we returned to the data of BPK282 cl4 and BPK081 cl8 to characterize the karyotypes that are present in each clone. In BPK282 cl4, the most frequent karyotypes were very similar to each other, diverging by copy number changes in one to three chromosomes when compared to the most frequent karyotype (kar1—Figure 2A). In BPK081 cl8, however, the nearly diploid kar1, which was present in 82% of the cells, and the two next most abundant karyotypes showed very different aneuploidy profiles, diverging by copy numbers of 8 to 10 chromosomes (Figure 2B). In addition, in both clones, the most frequent karyotype (kar1) is similar to the average aneuploidy profile of the respective parent strain from which each clone was derived (Figure 1A, B, left panel), suggesting that, in each clone, kar1 corresponds to the karyotype of the founder cell, and thus, the other karyotypes of each population arose from their respective kar1.

To develop a hypothesis of the karyotype evolution during expansion of both BPK282 cl4 and BPK081 cl8 populations, we built a dissimilarity network based on the number of chromosomes with different copy numbers between each karyotype found in each population (Figure 2C). Both populations of cells are at different stages of expansion (about 126 and 56 generations since cloning, respectively), but we observe in each of them a proportionally comparable number of somy changes events (steps in the network): (i) for BPK282 cl4, 514 steps/126 generations/1516 sequenced cells = 0.0027 and (ii) for BPK081cl8, 260 steps/56 generations/2378 sequenced cells = 0.002. However, distinct patterns are observed between both clones. In BPK282 cl4, the most frequent karyotypes (black nodes) are linked to each other by somy changes in only single chromosomes (black lines). Assuming kar1 as the founder of this population, almost every frequent karyotype can be traced back to it through cumulative single copy number alterations. In contrast, the network of BPK081 cl8 shows a very distinct pattern (Figure 2C). Here, the 3 most frequent karyotypes are distant from one another and lack single-step intermediates between them.

### High frequencies of polysomies are restricted to a specific group of chromosomes

**Figure 3.**
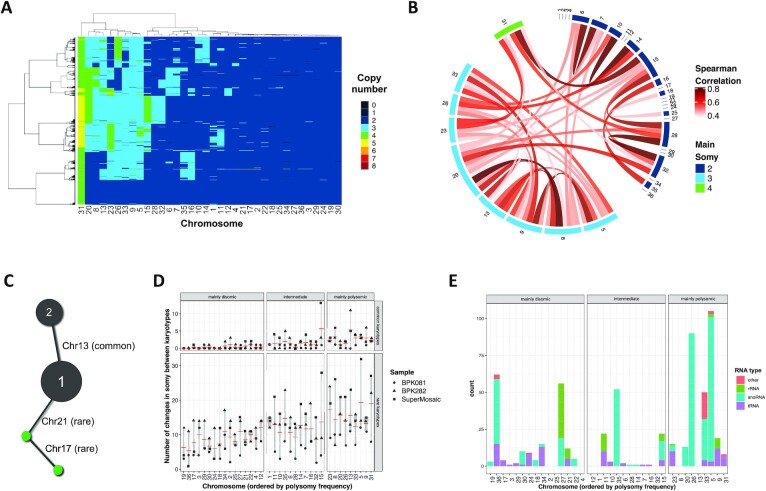
High frequencies of polysomies are restricted to a specific subset of chromosomes. (**A**) Heat map depicting the copy number of the 36 chromosomes across promastigotes from different clones/strains. Here, 251 promastigotes of each cluster of the mixed sample and from BPK282 cl4 and BPK081 cl8 are represented. Chromosomes are hierarchically clustered based on their somy values. (**B**) Chord diagrams representing the Spearman correlation between the copy number of all chromosomes. Only correlations higher than 0.4 and with p-value lower than 0.05 are represented. (**C**) Ilustration of the analysis done based on the karyotype networks of BPK282 cl4, BPK081 cl8 and the super mosaic population in order to quantify changes in somy for each chromosome across different karyotypes. In this image, kar1 and kar2 are found in more than two cells (black nodes), so they are considered common karyotypes. Here, chr13 is the only chromosome that displays a different somy between them, so this is considered as a somy change event for chr13. A second karyotype differs from kar1 by a change in somy in chr21. As this karyotype is found only in one cell, it is considered a rare karyotype. (**D**) Graph indicating the number of somy change events for each chromosome among the common karyotypes (found in two or more cells – top panel) or the rare karyotypes (found in only one cell - bottom) in the 3 samples submitted to SCGS. Chromosomes are divided in three groups: mainly disomic (found as disomic in more than 95% of the cells), intermediate (found as polysomic in more than 5% of the cells but less than 50%), and the mainly polysomic (found as polysomic in more than 50% of the cells). (**E**) Distribution of non-coding RNAs across *L. donovani* genome. Ribosomal RNAs (rRNA), small nucleolar RNAs (snoRNAs) and transporter RNAs (tRNAs) were identified based on the Rfam and TrypDB database.

We and others have demonstrated that high frequencies of polysomies were restricted to a specific subset of chromosomes when comparing the average aneuploidy of 204 *L. donovani* strains previously analyzed in bulk (23,29). To address if the same applies to single *Leishmania* cells, we created a diverse artificial population by randomly selecting and merging the data of equal numbers of single cells from BPK282 cl4 and BPK081 cl8 as well as from each cluster of the super mosaic population, assuming each cluster represents one of the mixed strains. In this artificial population, we observed that at least 15 chromosomes are consistently disomic in the vast majority of cells in a clone/strain-independent manner (Figure 3A). All these chromosomes also show an average disomy in most of the 204 strains mentioned above (Supplementary Figure S5A, B). These chromosomes are referred here as ‘mainly disomic’. Conversely, apart from the usually tetrasomic chr31, 8 chromosomes (chr5, chr8, chr9, chr13, chr20, chr23, chr26 and chr33) are found with 3 or more copies in most cells, again fitting with previous observations made on the 204 *L. donovani* strains (23,29). We call these chromosomes as ‘mainly polysomic’. A Spearman correlation test highlighted potential synchronies between the copy numbers of these chromosomes, with the strongest correlations being observed between chr20 and chr8, and between chr5 and chr9 (Figure 3B, Supplementary Figure S5C). There is also an association between the somies of chr31 and chr15, which is also noticeable in Figure 3A, where when the somy of chr31 increases to 5, the somy of chr15 also increases to three or even four copies, suggesting a potential dosage interplay between these chromosomes. Strong dosage correlations are also observed between chromosomes chr6 and chr7, chr10 and chr14, chr15 and chr28, chr16 and chr35, and between chromosomes chr28 and chr32. These chromosomes are usually found as disomic but still display a polysomy in a relatively large (>5%) subgroup of cells. We refer to these chromosomes as ‘intermediate’.

It is unclear whether (i) the disparity in the frequency of polysomies between chromosomes is due to intrinsic differences in the chances of overamplification of each chromosome along the expansion of the population (some chromosomes being specifically unstable) or (ii) if every chromosome has the potential to have its somy altered but the expansion of polysomies in a population is limited by selective pressures. Thus, we revisited the karyotype network of each population, including the ‘super mosaic’ (Supplementary Figure S5D), to investigate differences in dosage stability between chromosomes. In this analysis, we determined which were the chromosomes that were different between a karyotype and each other karyotype that was connected to it in the network. Thus, unstable chromosomes are expected to change more frequently between karyotypes. Connections between two karyotypes found in more than 1 cell were named as ‘common karyotypes’ while connections where at least one of the karyotypes was found in only 1 cell were marked as ‘rare karyotypes’ (Figure 3C). As expected, the 15 mainly disomic chromosomes display little, if any, alteration events in their copy numbers among the common karyotypes in all three samples (Figure 3D). However, between the rare karyotypes, all chromosomes are susceptible to somy alterations, although the mainly polysomic chromosomes still display a higher alteration frequency (*P*-value < 0.0001 – Supplementary Figure S5E). Interestingly, the mainly polysomic chromosomes chr20 and chr26 are among the most stable chromosomes between the common karyotypes but are still highly variable among the rare karyotypes. A similar trend is also observed for chromosomes chr5, chr23 and chr31, which could be an indicative of selective pressure limiting dosage changes in these chromosomes.

In order to investigate potential features specific to the mainly polysomic chromosomes that could be related to their higher frequency of polysomies, we performed a series of *in silico* enrichment analysis. Gene Ontology (GO) analysis did not reveal any obvious relationship between gene content and the prevalence of polysomies (Supplementary Figure S6). As the first analysis took into account only protein-coding genes, we turned our attention to non-coding RNAs, which could be relevant in this context, given the known high level of post-transcriptional regulation in *Leishmania*. Indeed, an in silico scan for non-coding RNAs (rRNA, tRNA, snoRNA and others) suggested an enrichment of small RNAs in some of the mainly polysomic chromosomes (Figure 3E), and especially for small nucleolar RNAs (snoRNAs), a statistical significant enrichment could be detected on mainly polysomic chromosomes (*P*-value < 0.001, Fisher's exact test). A significant number of hits for snoRNAs are mapped to chr5, chr26 and chr33, which are among the chromosomes with the most frequent polysomies, as well as chr35, which is trisomic in the majority of BPK282 cl4 cells and is also trisomic in the average aneuploidy of several *L. donovani* strains (29). Although preliminary, this observation suggests a potential relationship between the snoRNAs content of a chromosome and its prevalence of polysomies in cultivated promastigotes.

### SCGS reveals particular karyotypes among rare single cells

As shown above, kar2 and kar3 of BPK081 cl8 show a baseline diploidy, i.e. the majority of chromosomes are disomic, with 8–10 trisomic chromosomes and tetrasomy or even a pentasomy for chr31. However, we found in the same population 5 cells with a karyotype similar to kar2 and kar3, but in which the scaling algorithm (see supplementary text for details on how cells ploidies are determined) defined the baseline ploidy of their karyotypes as triploid (kar 13 and kar 35 in Figure 2B, Supplementary Figure S7). These cells also display a higher depth (0.49× on average) compared to the other cells considered diploid (0.29× on average), indicating they had a higher DNA content. Although it is not possible to rule out that these cells are in fact doublets, it is expected that a doublet between two diploid cells would yield either a diploid profile, as the ones identified in the doublets from the super-mosaic population (Supplementary Figure S8) or a tetraploid profile, similar to the ones observed in kar30 and kar32 for instance. However, scaling these cells to 2*n* or 4*n* yields raw somy values which are further away from integers than when they are scaled to 3*n* (Supplementary Figure S3C), indicating that the ratios between the depths of the chromosomes of these cells are more compatible with a 3*n* profile. Noteworthy, tetraploid karyotypes were not found in BPK282 cl4 and the only three cells identified with a potential baseline triploidy exhibited an aneuploidy pattern very distinct from any other karyotype in that population (Supplementary Figure S7).

**Figure 4.**
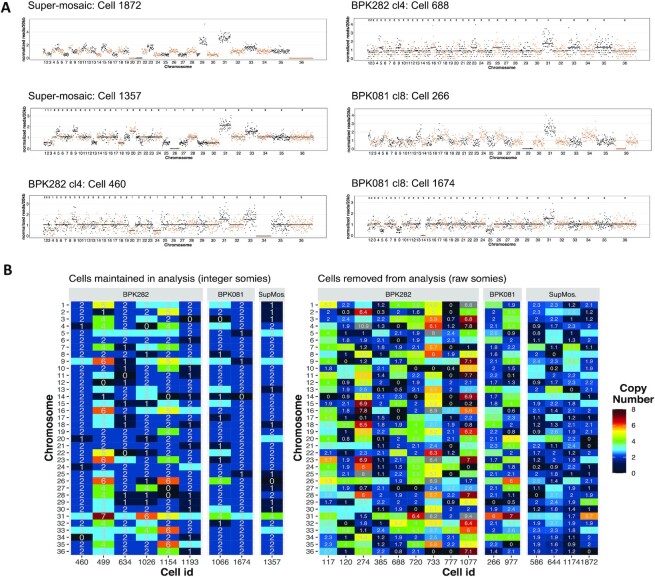
Cells with nullisomic chromosomes. (**A**) Example of cells displaying one or more nullisomic chromosomes. The dots represent the normalized read depth of each 20 kb bin. The integer somy values calculated for each cell are depicted in the top part of each box for cells that were not excluded from analysis. A black line shows the integer somy values divided by the cell's scale factor (Sc) for comparison. (**B**) Karyotype of all cells with at least one nullisomic chromosome identified in our SCGS data. Cells that were removed from analysis and therefore did not have their somy values converted to integers are separated in the right panel, displaying their raw somy values instead.

Moreover, within the BPK282 cl4 and BPK081 cl8 populations, we also observed rare cells displaying chromosomes with an estimated somy of 0 (nullisomy). The bam file of these cells showed that no reads were mapping to these chromosomes in sharp contrast with other chromosomes of the same cell, suggesting that in these cells, these chromosomes were absent (Figure 4A). Nullisomic chromosomes were found in all the populations sequenced here: among which, 4 in BPK081 cl8 (0,15% of the sequenced cells) and 15 from BPK282 cl4 (0,88%). Moreover, the aneuploidy profile of these nullisomic cells was not similar to any other karyotype identified in each sample (Figure 4B). Partial chromosome deletions were also observed, as for instance in chr13 and chr36 of the cell 688 from BPK282 cl4, in the chr36 of the cell 266 from BPK081 cl8.

## DISCUSSION

Cellular heterogeneity is increasingly implicated as one of the major sources of adaptative potential for unicellular pathogens (42,43). We explored here a specific manifestation of this phenomenon, i.e. mosaic aneuploidy, in a unique model, *Leishmania*. By applying a high throughput SCGS method, we could determine for the first time the complete karyotype of thousands of individual *Leishmania* cells from two distinct clonal populations *in vitro*. We found a high level of mosaic aneuploidy, affecting essentially the same, limited subset of chromosomes. We explored the evolution of mosaicism in both populations, starting from two distinct founder karyotypes, one nearly euploid and another highly aneuploid.

The present SCGS study allowed us to evaluate and extend hypotheses on mosaic aneuploidy in *Leishmania* previously based on FISH measurements (3,26). Although some divergencies were observed here between FISH and SCGS, our data are in agreement with most predictions. Accordingly, mosaic aneuploidy was confirmed in all populations sequenced here, and karyotypes frequency distributions, in particular for BPK282 cl4 clone (208 karyotypes among 1516 cells), were similar to the distribution predicted with FISH data obtained for seven chromosomes of a long-term cultivated *Leishmania major* population (∼250 karyotypes in ∼2000 cells—Sterkers *et al.*, 2012 Figure 4). Similarly, the observation of nullisomic chromosomes by SCGS supported previous FISH analysis of dividing nuclei: this showed that for both chromosomes 2 and 22 of *L. major*, around 1% of the evaluated parasites were displaying a ‘1 + 0’ distribution of chromosomes between daughter cells (3). In BPK081 cl8, proportionally fewer karyotypes were identified compared to BPK282 cl4, which might be a consequence of either a reduced tendency of the founder diploid karyotype to somy alterations and/or due to the fact this clone was at an earlier stage of expansion *in vitro* (∼56 generations, compared to the ∼126 generations in BPK282). Indeed, when normalizing the number of karyotypes, similar values were observed for both clones: respectively 10exp^–4^ and 9exp^–4^ new karyotypes/generation/sequenced cell.

Our SCGS data, however, do not corroborate the previous assumptions that all chromosomes are found with at least two somy states (3,26), as high levels of somy variation were restricted to a subset of chromosomes in our experimental conditions. Noteworthy, this feature could be species-specific as the analysis of single *L. braziliensis* cells in similar conditions of in vitro maintenance showed a generalized trisomy of chromosomes chr11 and chr25 (27), while these two chromosomes were disomic in the majority of *L. donovani* cells here studied. We also observed a higher tendency of FISH to report trisomies and monosomies in chromosomes which were defined by SCGS as mostly disomic in almost all cells of BPK282 cl4 and BPK081 cl8 clones, as chr01 and chr22. This discrepancy is likely due to accuracy limitations in FISH (44,45).

The SCGS data reported here also allowed us to draw some hypothesis regarding the origin and evolution of mosaic aneuploidy *in vitro*. We have previously demonstrated that intracellular amastigotes sequenced directly from patient samples usually display a diploid average aneuploidy similar to the profile of the BPK081 cl8 clone, although variations in somies were observed in some samples (46). However, when these amastigotes were isolated from patients or experimental animals and transformed to promastigotes *in vitro*, in most cases they progressively evolve towards highly aneuploid profiles (19,46,47). Thus, the 2 clones here studied provide complementary models to understand the dynamics of the emergence of mosaic aneuploidy *in vitro*; BPK081 cl8 which founder karyotype had the diploid profile, representing an early stage of expansion in culture; and BPK282 cl4, which founder karyotype was already highly aneuploid (likely kar1), representing later stages.

In the BPK081 cl8, a minority of highly aneuploid subpopulations were observed, contrasting with the founder diploid karyotype (kar1), indicating that at early stages of clonal expansion in culture, the evolution of mosaicism starts with drastic changes in karyotypes, in this case the observed changes in somy of 8 to 10 chromosomes leading to highly aneuploid cells (kar2 and kar3). These drastic changes in somies could occur through cumulative small steps, i.e., somy alterations in single chromosomes at each cell division, followed by fixation and further expansion of the fittest aneuploidies and loss of intermediate links between these karyotypes during clonal evolution. Alternatively, kar2 and kar3 in BPK081 cl8 may have originated independently from kar1 by simultaneous amplifications of multiple chromosomes. However, the presence of potentially triploid cells which resemble kar2 and kar3 opens other possibilities. On one hand, polyploidization has been demonstrated as an important mechanism in yeasts for quickly generating multiple and highly discrepant aneuploid karyotypes from a single parent through assorted mis-segregation of chromosomes during downstream cell divisions (9). In case a similar mechanism occurs in *Leishmania*, these 3n karyotypes found in BPK081 cl8 could represent an intermediate step between whole genome polyploidization event and reversion to aneuploid kar2 and kar3. On the other hand, 3n karyotypes could be reminiscent of hybridization, which was recently shown to occur *in vitro* (48); the common observation of 3n karyotypes in *Leishmania* after hybridization in sand flies supports this hypothesis (49–52). Further work is required in order to test these hypothesis.

Surrounding the three major karyotypes in the network of BPK081 cl8, other minor karyotypes with single somy alterations are observed, suggesting that once a successful karyotype expands, small variations of it are continuously generated by small changes in somies. This pattern is more evident in the karyotype network of BPK282 cl4, where almost all karyotypes which are found in at least two cells are at one somy change distance from another karyotype, suggesting that these karyotypes were also continuously generated by cumulative steps of small somy alterations. Accordingly, the founder karyotype of this clone (likely kar1) was already highly aneuploid and well adapted to culture, as the parent population from which BPK282 cl4 was isolated was already in culture for 21 passages (Supplementary Figure S1).

Karyotypes displaying several polysomies are observed in most *in vitro* cultured *Leishmania* promastigotes analysed so far in bulk (29,53,54). This usually affects a specific group of chromosomes, largely overlapping with the 8 mainly polysomic chromosomes described here. The early amplifications reproducibly observed in the average aneuploidy profile of parasite populations in transition from *in vivo* to *in vitro* (46,47) suggest an adaptative role for specific polysomies in adaptation to culture. However, the mechanisms that determine which chromosomes are amplified are still poorly understood.

By investigating which chromosomes were more prone to somy alterations in rare and common karyotypes, we gathered evidence suggesting that all chromosomes can be stochastically amplified during population expansion, potentially at different rates, but we hypothesize that selective forces likely dictate the higher frequency of polysomies observed in some chromosomes. Changes in the average chromosome copy numbers of cell populations are directly reflected in the average amount of transcripts encoded by the genes present on these chromosomes (19,23) and to a certain degree also affect the average amount of certain proteins (24). Consequently, aneuploidy might lead to dosage imbalances between the product of genes located in chromosomes that display different somies. The frequently observed co-modulation of multiple chromosomes—estimated with Spearman correlations with single-cell resolution here and across 204 *L. donovani* isolates, in bulk, as previously described (23)—might reflect a dynamic compensation mechanism that reduces these imbalances and at the same time increases the dosage of key genes. Our GO analyses did not reveal any enrichment of biological functions in the (co-) amplified chromosomes. However, we observed an enrichment of snoRNA genes in some of the mainly polysomic and intermediate chromosomes, accordingly chr05, chr26, chr33 and chr35. This class of small RNAs is involved in the extensive processing of ribosomal RNA (rRNA) characteristic of trypanosomatids, directly affecting ribosomal biosynthesis and ultimately translation, both increased in cultured promastigotes (55,56). Amplification of these chromosomes as seen in many cells *in vitro* might ultimately boost the translation capacity of the cells due to a consequent higher abundance of snoRNAs. At the time of submission of the present article, a pre-print of a study was published where the role of posttranscriptional regulation in *Leishmania* fitness gain was examined (57). In this manuscript, snoRNAs are postulated to play a key role in differential regulation of mRNA stability through change in the composition of ribosomes. Specifically, differential expression of snoRNAs was shown to be correlated with changes in rRNAs epigenetic modifications, which were proposed to result in formation of fitness-adapted ribosomes. Consequently, by regulating the composition of ribosomes, the expression of beneficial and deleterious gene dosage effects could be regulated through translational control. Amplification of chromosomes carrying these snoRNAs as seen in many cells in vitro might ultimately alter and adapt the translational capacity of the cells due to a consequent higher abundance of snoRNAs as postulated by Spaeth *et al.*

The high diversity of karyotypes identified in both models here described is in agreement with the idea of mosaic aneuploidy being a constitutive feature in *Leishmania* (25). Knowing that average aneuploidy (as defined by bulk genome sequencing) is much lower and different *in vivo* than *in vitro* (19), further work would be required to compare the extent and nature of mosaicism *in vitro* and *in vivo*. The generation of karyotypic heterogeneity represents a source of functional diversity, due to variations in genes dosage (19), and it is also expected to facilitate the removal of detrimental mutations and the fixation of beneficial haplotypes (23,26). Although in a given environment some very different karyotypes might be limited to low frequencies, they may provide to the population a major (pre-)adaptation potential to unpredictable environmental changes, such as a change of host or drug pressure associated to chemotherapy (19,21,22,58). Time-lapse SCGS studies of populations of parasites during clonal expansion under stable or varying environments are needed to monitor the dynamics of mosaicism and test this pre-adaptation hypothesis. Combining SCGS with single-cell transcriptomics could also allow to understand better the impact of gene dosage imbalance on transcription with a single-cell resolution. Thus, high throughput single-cell sequencing methods represent a remarkable tool to understand key aspects of *Leishmania* biology and adaptability.

## DATA AVAILABILITY

Raw sequencing data have been deposited in BioProject (NCBI) with accession number PRJNA720894. Custom scripts used in the present study are available at GitHub (https://github.com/gabrielnegreira/scgs-somy).

## Supplementary Material

gkab1203_Supplemental_Files
