# Supplementary material for: High throughput single-cell genome sequencing gives insights into the generation and evolution of mosaic aneuploidy in Leishmania donovani
Source: Nucleic Acids Res. 2021 Dec 10;50(1):293–305. doi: 10.1093/nar/gkab1203 (PMC8886908; doi:10.1093/nar/gkab1203)
Supplement: gkab1203_Supplemental_Files [file gkab1203_Supplemental_Files.zip › Negreira_GH_et_al_2021_-_Supplementary_data_-_Rebutal.docx]

Supplementary text to

**High throughput single-cell genome sequencing gives insights into the generation and evolution of mosaic aneuploidy in *Leishmania donovani***

by

Gabriel H. Negreira^1^, Pieter Monsieurs^1^, Hideo Imamura^1^, Ilse Maes^1^, Nada Kuk^2^, Akila Yagoubat^2^, Frederik Van den Broeck^1^, Yvon Sterkers^2^, Jean-Claude Dujardin^1,3^, Malgorzata A. Domagalska^1^

**Table of Contents**

[Glossary 2](#_Toc86048770)

[Supplementary methods 2](#_Toc86048771)

[Single-cell DNA sequence data analysis 2](#_Toc86048772)

[Doublet detection 5](#_Toc86048773)

[Supplementary results & discussion 5](#_Toc86048774)

[Sequencing statistics 5](#_Toc86048775)

[Estimation of the frequency of doublets 6](#_Toc86048776)

[Gene Ontology analysis 7](#_Toc86048777)

[Supplementary References 9](#_Toc86048778)

[Supplementary Figures 10](#_Toc86048779)

# Glossary

| Term | Definition |
| --- | --- |
| Bulk Genome Sequencing | Whole genome sequencing performed in a group of cells combined as a single sample. |
| Single Cell Genome Sequencing (SCGS) | Genome sequencing performed in single cells individually. |
| Somy | The number of copies of a given chromosome in a cell. |
| Polysomy | A somy higher than 2. |
| Karyotype | The set of copy numbers of all chromosomes in a cell. |
| Cell Karyotype | The karyotype of a cell determined by SCGS. |
| Populational Karyotype | The average karyotype of a population determined by bulk genome sequencing. |
| Ploidy | The most frequent somy in a karyotype. |
| Euploidy | A condition where all chromosomes display the same somy in a cell. |
| Aneuploidy | A condition where one or more chromosomes display a somy that diverges from the other chromosomes in the same cell. |
| Mosaic Aneuploidy | A condition where different aneuploid karyotypes co-exist in the same population. |
| Cell scale factor | The lowest number between 1.8 and 5 by which when the average normalized read depths of all chromosomes in a cell are multiplied the resulting numbers are the closest to integers as possible. |
| Raw somy | The average normalized read depth of a chromosome multiplied by the cell’s scale factor. |
| Integer somy | The integer value assigned to a raw somy. |

# Supplementary methods

## Single-cell DNA sequence data analysis

Illumina Base call files (BCL) were demultiplexed and converted to FASTQ files using the cellranger-dna mkfastq command of the CellRanger^TM^ DNA pipeline (10X Genomics). The FASTQ files were then used as inputs to the cellranger-dna cnv command in order to associate reads to individual cells based on their 10X barcodes and to map reads to a customized version of the LdBPKv2 *L. donovani* reference genome (available at <ftp://ftp.sanger.ac.uk/pub/project/pathogens/Leishmania/donovani/LdBPKPAC2016beta/>), where ‘N’s were added to the ends of chromosomes 1 to 5 to reach the 500kb minimum size allowed by the CellRanger DNA pipeline. The pipeline divides the genome into adjacent 20kb bins and outputs a CSV file containing the number of reads mapped to each bin. This file was used to estimate chromosomes copy number in a custom script written in R.

An overview of the steps performed by the script is shown in supp. fig. 3A. The script first removes bins with a low number of mapped reads by eliminating any bin showing an average depth of 0.5 read/cell. Then, the difference between the median number of reads of each bin and the chromosomal median is calculated. Bins with outlier values are determined using the boxplot.stats function from the R package grDevices v3.6.2. These outlier bins are removed from downstream analysis (supp. fig. 3B). This also excludes common local-CNVs found in some *L. donovani* strains, as for instance the H-Locus and the M-Locus in Chr23 and Chr36 respectively (2), present in the BPK strains/clones but absent in the HU3 strain. After removal of outlier bins, the bins depths are normalized by the cell mean and are used to estimate intrachromosomal variation (ICV). ICV is determined for each cell by dividing each chromosome in 3 segments and calculating the ratio between the segment with the highest and the segment with lowest depth. The mean of the five highest ICV values (i.e. the 5 most variable chromosomes in a cell) is assigned as its ICV-score. The distribution of ICV-scores in each sample was graphically analyzed in order to determine a threshold for exclusion of noisy cells. This threshold was defined as 2.0 for BPK282 cl4 and 1.7 for the BPK081 cl8 and the ‘super-mosaic’ samples.

The copy number of chromosomes in a cell is defined based on their normalized mean depth (NMD), i.e., the mean of the normalized depth values of the 20kb bins of a chromosome. In this sense, NMDs reflects the relative differences in copy number between chromosomes, but absolute copy numbers must be inferred based on the ratios between NMDs of different chromosomes in a cell. Thus, considering that chromosomes copy numbers must be integers, the script uses an approach to determine absolute copy numbers which consists of multiplying NMDs by a scale factor which minimizes distances between the multiplied NMDs and integers. Therefore, the scale factor is defined as the lowest value between 1.8 and 5 which results to the closest approximation of NMDs to integers when they are multiplied by this factor. As the scale factor is directly affected by the ploidy of the cell, the limitation of the scale factor to values higher than 1.8 heuristically assumes that the lowest baseline ploidy of a cell is 2n. This was done to prevent that 2n cells with no odd somy value would be scaled as 1n cells.

In order to determine the scale factor, the script multiply the NMDs of a cell by 1000 equidistant numbers between 1.8 and 5. For each multiplication, the difference between the resultant values and their closest integers is calculated for each chromosome and averaged. The value that results in the lowest average distance to integers is then assigned to the cell as its scale factor (supp. fig. 3C). In case two or more scale factors result in the same average distance to integers, the one with the lowest value is chosen.

Since *Leishmania* chromosomes are biased in GC content (3), with small chromosomes (Chr1 to Chr5) displaying a higher GC content than others, amplification bias due to differences in GC content can have a negative impact in the determination of the copy number of these chromosomes. Plotting the distribution of NMD values leads to different peaks, each peak representing one of the somy values, however, the peaks of these small chromosomes with high GC content are shifted relative to the other chromosomes (supp. fig. 3D upper panel). Thus, to compensate for chromosome-specific amplification and to further define the somies of the cells, the above explained scale factor are used at two levels, i.e., at population level (all cells combined) as well as at single cell level (defined for each cell). In this sense, the script first defines a single scale factor to the whole population (Sp) by which NMDs are multiplied and the distribution peak of the scaled NMDs of each chromosome is adjusted to the closest integer (supp. fig. 3D bottom panel). Then, these values are divided back by Sp and based on this output a second scale factor is defined for each cell (Sc). Thus, the NMDs of the chromosomes in a cell after bias compensation multiplied by the cell’s Sc defines the ‘raw somies’ of the chromosomes of that cell.

Despite the fact that the abovementioned steps have moved the NMDs distribution closer to integer values, those values are still floating-point numbers. To determine the cells karyotypes, the raw floating-point somies are converted to integer copy numbers using Gaussian Mixture Models (GMMs). To generate a GMM for each chromosome, a vector containing all raw somy values determined for that chromosome among the filtered cells in a sample is used as input to the normalMixEM function of the mixtools R (4), following the defined rules bellow:

1. The possible integer values are defined as the number of different integers found when all values in the vector are rounded to the closest integer.
2. The number of components (k) is determined as the total number of possible integer values.
3. The ratio between means (µ) of k gaussians are constrained to the ratios between the possible integer values.
4. If for a given gaussian, less than 5% of the values are inside the interval between µ-0.2 < µ < µ+0.2, the standard deviation (σ) of that gaussian is arbitrarily limited to 0.1.
5. At least 5 iterations must be performed before a gaussian is defined.

Thus, for each chromosome in a sample, a gaussian is built for each possible integer somy (supp. fig. 3E). Raw somies are then converted to the rounded µ of the gaussian of which they have the higher probability of belonging to. Since the GMMs must be built between cells sharing the sample baseline ploidy, and as the vast majority of cells in all samples sequenced in the present study had a scale factor lower than 2.5 and consequently were considered 2n cells (supp. fig. 3F), the GMMs were applied only to 2n cells. Moreover, since the number of non-2n cells were always very low, GMMs could not be built separately for cells with other baseline ploidies. Thus, cells which baseline ploidy was different than 2 were treated differently. In this case, cells with intermediate somies, i.e, with at least one raw somy values that are at a distance greater than 0.4 from its closest integers, were considered unresolvable and were removed from downstream analysis. The reminiscent had their raw somy values simply rounded to the closest integer. Karyotypes were then defined as the concatenated set of integer somy values found in a cell.

## Doublet detection

Two different methods were used for doublet detection, i.e. an in-house developed methodology and Demuxlet (5), both exploiting the difference in SNP profile between HU3 cells versus other cell lines.

The in-house developed approach uses the following methodology: 1) Homozygote SNPs for the HU3 strain are predicted based on the genome of the HU3 strain sequenced by BGS (data not shown). 2) For each of those HU3 homozygote SNPs, the occurrence of this SNP is derived for each of the single cells in the 4-strains mixture sample (further called ‘super-mosaic’). Given the low sequencing depth per cell (on average around 1x), this will report the absence or presence for each SNP. 3) For the HU3 cells in the super-mosaic, the majority of SNPs should be detected, while for the other three strains no SNPs should be detected. In case of a doublet consisting of a HU3 cell with a cell from one of the other three strains, two different scenarios can occur: If the sequencing depth is low, only the allele of one of the two cells can be predicted, while in case of a sufficient sequencing depth (at least 2x), both alleles (either the HU3 or the reference allele) can be detected, resulting in an allele frequency of 50%. In both cases, overall detection rate of the homozygote SNPs should be around 50%. In order to compensate for sequencing errors and differences in sequencing depth, libraries detecting between 10% and 90% of the HU3 homozygote SNP list were classified as doublets. Homozygote SNPs where predicted based on the genome of the HU3 strain. Genetic variants were detected using the mpileup and call command of BCFftools (version 1.10.2). The view and query command of BCFtools were used to filter out genetic variants fulfilling the following conditions 1) minimum sequencing depth of 100, 2) only SNPs i.e., removing indels, 3) biallelic, 4) homozygous. In a second step, for each single cell those SNP positions are checked using the bcftools mpileup command.

Demuxlet was run using the default parameters with the following input: 1) the bam file returned by the Cell Ranger software, produced for the single-cell experiment with the super-mosaic, 2) a vcf file describing the two different SNP profiles, i.e., the SNP profile for HU3, and the SNP profile for the three other strains.

# Supplementary results & discussion

## Sequencing statistics

Summary of sequencing statistics is provided in table S1. The BPK282 cl4 and BPK081 cl8 were sequenced with the same targeted depth (75.000 reads per cell) but BPK282 cl4 sample displayed a depth which was lower than anticipated (29.192 reads per cell). This was due to a high fraction (53.3%) of reads without a cell barcode in this sample, which according to the manufacturer indicates free floating DNA or a problem during library prep, but which unlikely affect copy number estimation. The scCNV library of the super-mosaic sample was sequenced deeper (209.000 reads per cell) to better allow the distinction between doublets. Higher coverage depths per cell were also associated with lower intra-chromosomal variation and lower frequency of intermediate somy values (supp. fig. 9A-B). This explains why sample BPK282 cl4 displayed a higher overall ICV score compared to the other samples.

The noisy nature of whole genome amplification ultimately leads, in some cases, to the existence of raw somy values which are at similar distances from two integers. Although the conversion of raw somies into integers could be achieved by simply rounding the raw somy values to the closest integers, this could overestimate the number of karyotypes identified in a population, as the wrong determination of a somy value of a single chromosome in a single cell is sufficient to lead to a new artificial karyotype. Thus, in order to convert the raw somy values into integers, we used a more stringent approach by constructing GMMs based on the distribution of raw somy values of each chromosome among cells in a given sample. One of the consequences of using this approach is that the frequency of which an integer somy value is present in a population influences the probability of a raw somy value to be assigned to this integer. This favors that intermediate somy values are assigned to the most frequent integer somy values in the population, reducing the chances of misinterpreting an intermediate value as a new, rare integer, and consequently greatly reducing the number of artificial karyotypes caused by the misinterpretation of a somy. This is evident, for example, when comparing the number of karyotypes identified in the BPK282 cl4 sample using the GMMs (207 karyotypes) and when raw somies are just rounded to their closest integers (525 karyotypes - supp. fig. 9C).

Noisy data had also an impact on the scaling of the NMDs of cells into raw somies, as differences between chromosomes NMDs becomes less discrete. In the 3 samples submitted to SCGS here we noticed a higher ICV-score in a large fraction of cells which were scaled to baseline ploidies different than 2 (supp. fig. 9D). These cells were removed from karyotype estimation either due to their ICR-score being above the threshold, or due to the presence of unresolvable intermediate somy values as described in the supplementary materials and methods.

## Estimation of the frequency of doublets

The ‘super mosaic’ population was also used to estimate the frequency of doublets, i.e, the inclusion in a single droplet of two or more cells sharing the same 10X barcode, which was Based on the SNP profile of the HU3 line, each dataset with the same barcode containing either none of the HU3-specific SNPs (< 5% of the SNPs), or almost all of the HU3-specific SNPs (> 95% of the SNPs) were defined as singlets, while doublets contained a mixture of HU3-speficic SNPs and positions resembling the reference genome. Using this approach, from the 293 cells that were predicted as HU3 based on their SNP profile (including cells removed from karyotype estimation), 21 were predicted as doublets (supp. fig. 5), with a detection rate of SNPs varying between 14% and 58%. Since doublets formed by two HU3 cells would still be defined as a singlet and given that HU3 cells correspond to 15,4% of the population, we assumed that the 21 detected HU3+BPK doublets correspond to 84,6% of the total number of doublets containing an HU3 cell. Thus, we estimate that there are ~4 (the extra 15,4%) additional HU3+HU3 doublets, resulting in a total of 25 doublets. Extrapolating this fraction of 25 out of 293 HU3 cells to the whole single-cell population would correspond to a relative fraction of doublets of 8,53%, a frequency which is higher than anticipated for mammal cells according to the manufacturer’s guidelines (~1,4%). From the 21 detected doublets, 3 were originally removed from karyotype estimation due to high intra-chromosomic variation, and 6 displayed a karyotype that was also found in other cells. However, 11 karyotypes were exclusively found in one of the detected doublets (supp. fig. 5), indicating that a fraction of the low-occurrence karyotypes might be artifacts due to doublets.

## Gene Ontology analysis

Gene ontology (GO) analysis was performed using two approaches. We first applied an unsupervised GO analysis to look for enrichment of biological functions in the mainly polysomic chromosomes. However, no obvious relationships between chromosomal gene content and prevalence of polysomies could be found (supp. fig. 6A). We then tried a supervised approach. Since highly aneuploid karyotypes are more frequently observed in *in vitro* promastigotes than in amastigotes, we reasoned that the amplification of these chromosomes might affect pathways related to the promastigote stage. Thus, we selected enriched GO classes which were obtained from a previously published study in which we studied differential expression between promastigote and amastigote cell cultures (19). The distribution of the corresponding genes on the mainly polysomic chromosomes was compared to the distribution on chromosomes with a stable disomy. However, this approach also did not disclose biological functions located on the amplified chromosomes (supp. fig. 6B).

# Supplementary References

1. Baker,J.R., Brown,K.N. and Godfrey,D.G. (1978) Proposals for the nomenclature of salivarian trypanosomes and for the maintenance of reference collections. *Bull. World Health Organ.*, **56**, 467–480.

2. Downing,T., Imamura,H., Decuypere,S., Clark,T.G., Coombs,G.H., Cotton,J.A., Hilley,J.D., De Doncker,S., Maes,I., Mottram,J.C., *et al.* (2011) Whole genome sequencing of multiple Leishmania donovani clinical isolates provides insights into population structure and mechanisms of drug resistance. *Genome Res.*, **21**, 2143–2156.

3. Imamura,H., Monsieurs,P., Jara,M., Sanders,M., Maes,I., Vanaerschot,M., Berriman,M., Cotton,J.A., Dujardin,J.C. and Domagalska,M.A. (2020) Evaluation of whole genome amplification and bioinformatic methods for the characterization of Leishmania genomes at a single cell level. *Sci. Rep.*, **10**, 1–13.

4. Benaglia,T., Chauveau,D., Hunter,D.R. and Young,D.S. (2009) Mixtools: An R package for analyzing finite mixture models. *J. Stat. Softw.*, **32**, 1–29.

5. Kang,H.M., Subramaniam,M., Targ,S., Nguyen,M., Maliskova,L., McCarthy,E., Wan,E., Wong,S., Byrnes,L., Lanata,C.M., *et al.* (2018) Multiplexed droplet single-cell RNA-sequencing using natural genetic variation. *Nat. Biotechnol.*, **36**, 89–94.

# Supplementary Figures

**Supplementary figure 1 – Flow chart of the two clonal populations used in the present study.** For BPK282 cl4, SCGS and FISH were performed in cultures at the same passage number. BGS was performed previously at passage 13 after cloning. For BPK081 cl8, all experiments were performed with the same culture. Number of generations is roughly estimated as 26+((p-1)*5)), where p is the number of passages. This is done assuming that it takes about 26 generations to reach a total of ~7x10^7^ cells starting from 1 cell, an approximation to the total number of cells usually found in a culture flask with 5mL of culture medium at the moment the first passage is done, and also assuming that each subsequent passage represents ~5 generations.


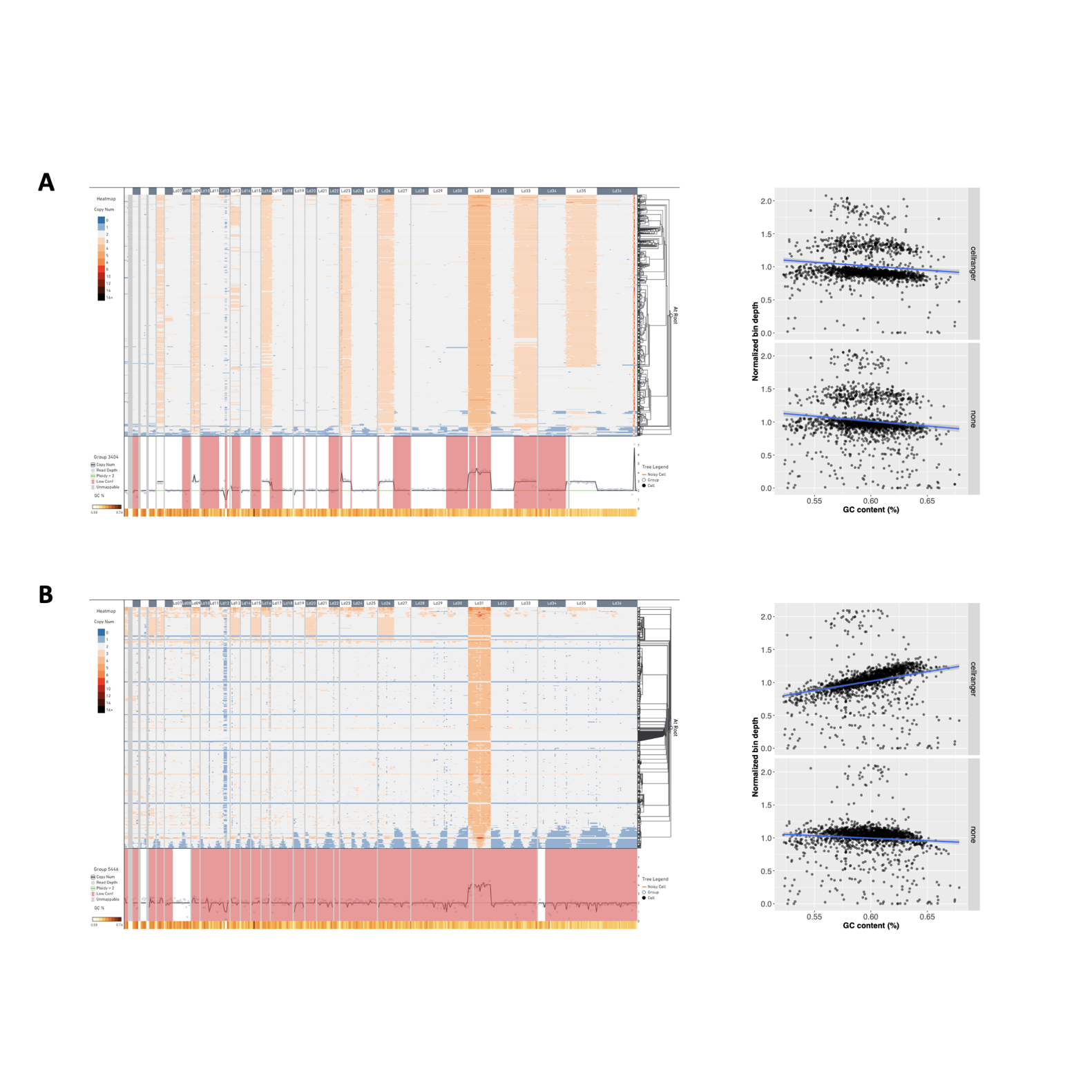


**Supplementary figure 2** - CNV profile of BPK282 cl4 (A) and BPK081 cl8 (B) calculated with the Cell Ranger^TM^ pipeline and visualized with the Loupe^TM^ scDNA Browser software (10X Genomics). In each sample, cells (rows) are arranged in 512 clusters, the maximum number of clusters allowed by the software. CNVs (columns) are depicted in windows of 80kb. Right panels display the effect of the the GC bias correction algorithm of the Cell Ranger^TM^ pipeline on the normalized read depth of bins (top) when compared to no bias correction (bottom).


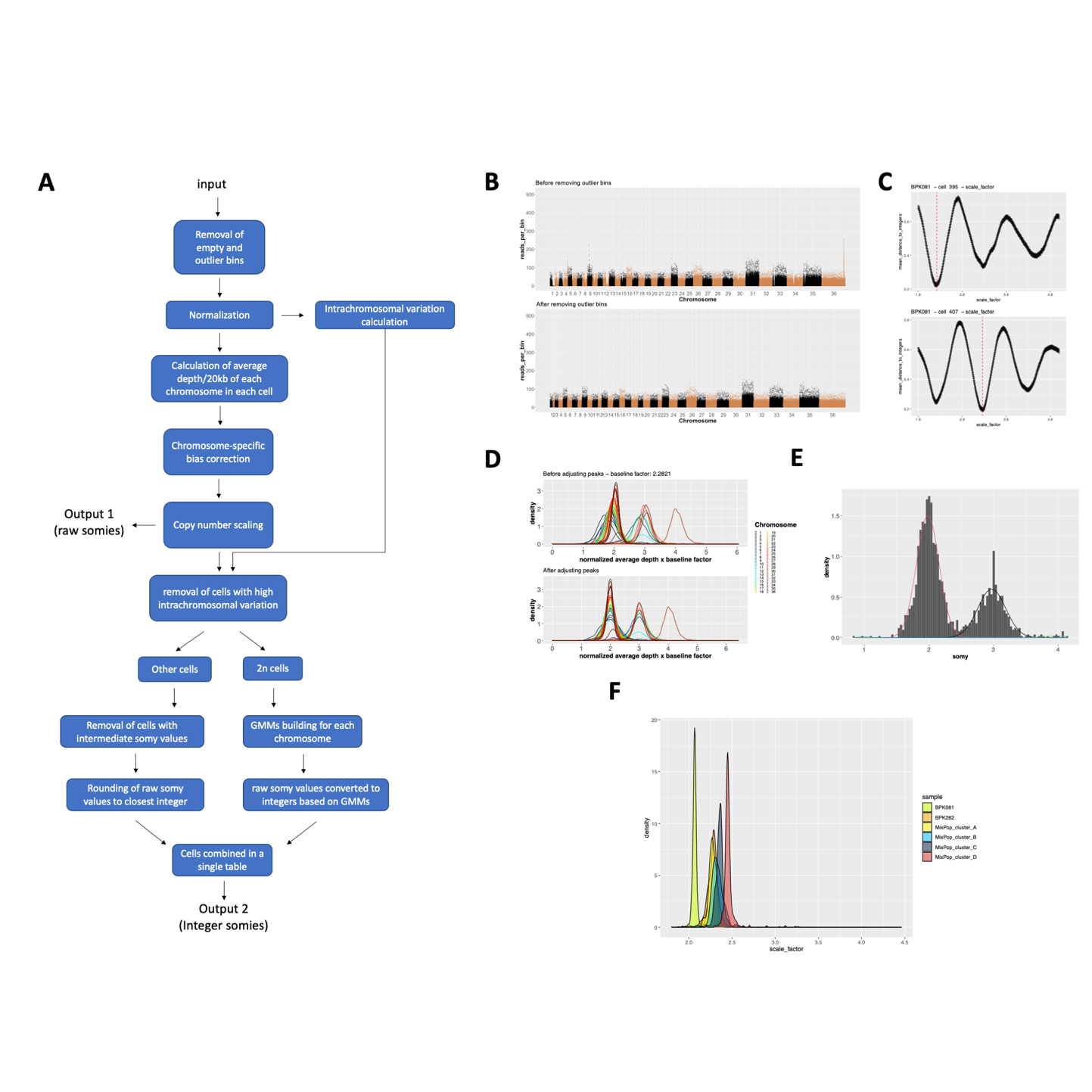


**Supplementary figure 3 -** Bioinformatics pipeline for somy estimation. **A.** Flow chart of the script developed to estimate chromosomes copy numbers based on their average depth/20kb bin. The input file is a matrix containing the read count of each 20kb bin for each cell. Two output files are generated, one with the raw somy values (floating points) and another with integer somy values. **B.** An example of the effect of the removal of empty and outlier bins in the BPK282 cl4 data. In this step, small intrachromosomal CNVs are also removed. **C.** Example of the determination of the scale factor for a 2N cell in the BPK081/0 cl8 sample with karyotype 2 (top panel) and a 3N cell with karyotype 13 (bottom panel). Y-axis represents the mean distance to integers when the NMDs of that cell are multiplied by a given scale_factor (x-axis). Red dashed line denotes the scale factor value defined for that cell. **D.** An example of the chromosome-bias correction step in the BPK282 cl4 data. **E.** Example of a Gaussian Mixture Model (GMM) built for chromosome 13 in the BPK282 cl4 data. The histogram represents the distribution of raw somy values for this chromosome in this sample, while the gaussian curves represent the GMM built for it. In this step, a gaussian is built for each integer, and raw somy values are assigned to the integer corresponding to the gaussian to which they have the higher probability. **F.** Distribution of the scale factors between all cells sequenced in this study.


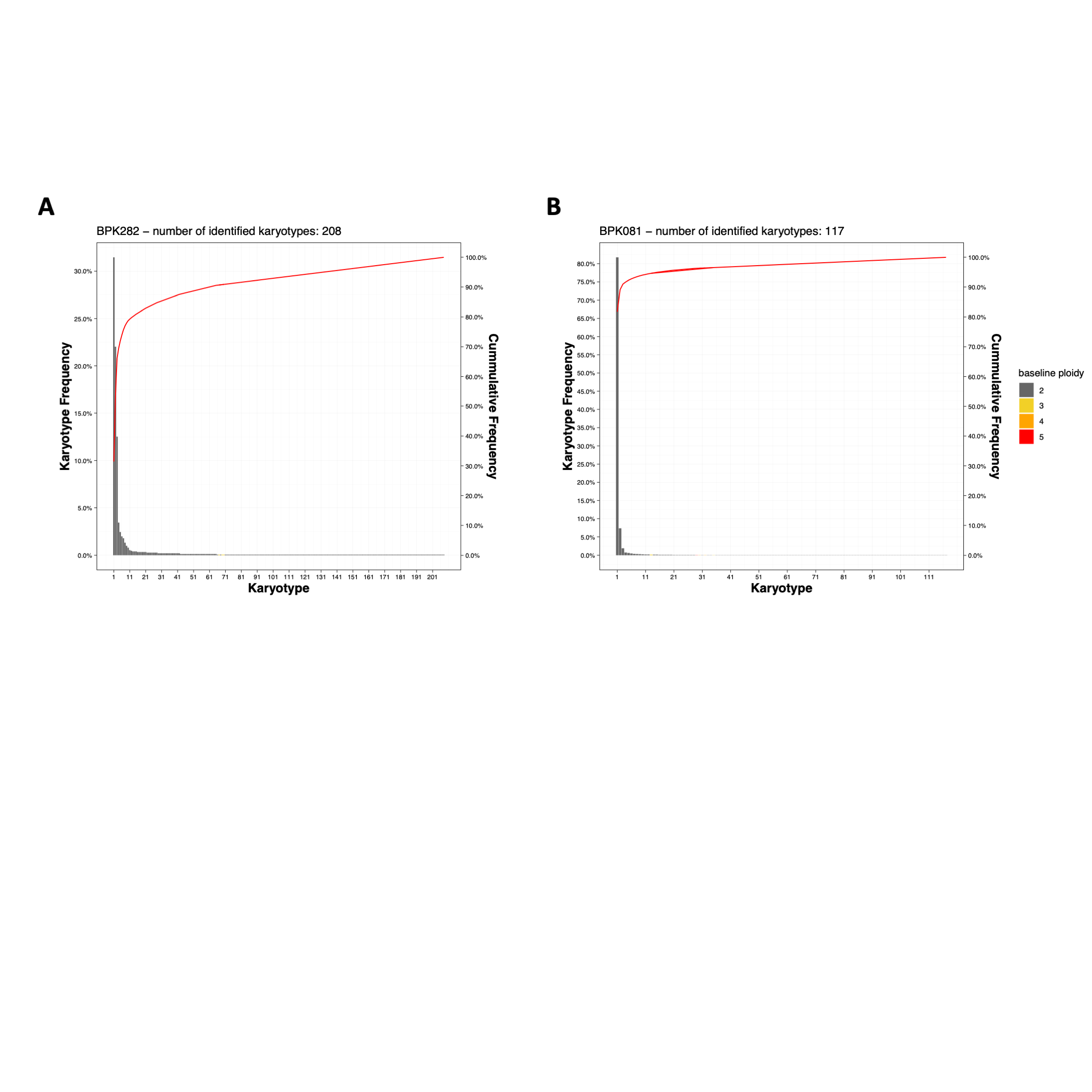


**Supplementary figure 4** - Frequency distribution of the karyotypes identified in **A.** BPK282 cl4 and **B.** BPK081 cl8 clones.


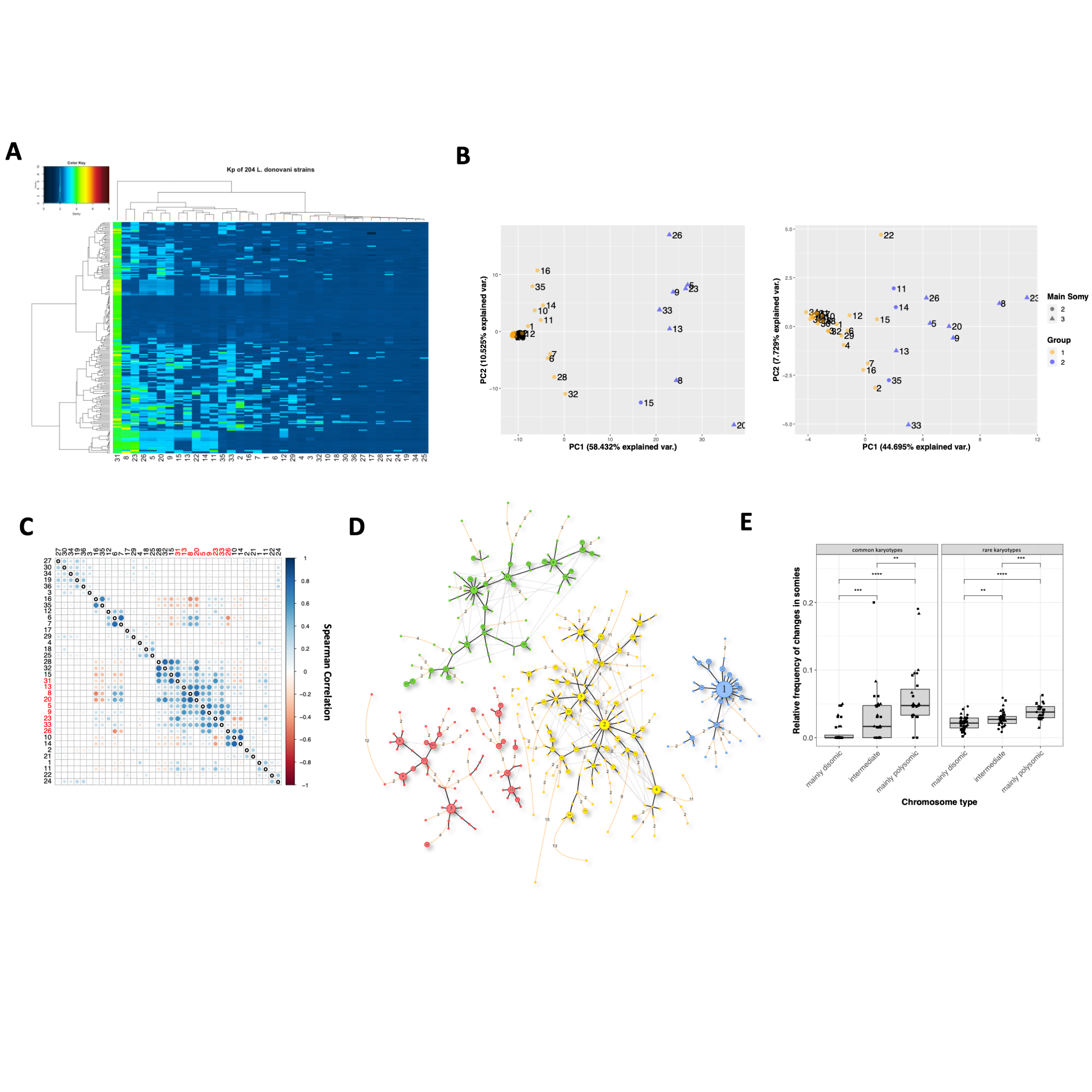


**Supplementary figure 5** – Supporting images for Fig 3 in the main text. **A.** Average Somies observed in the BGS data of 204 *L. donovani* strains (rows) with chromosomes (columns) hierarchically clustered. Data from Imamura H. et al, 2016. **B.** Principal Component Analysis constructed based on the somy values of each chromosome (dots) found in the 1554 cells from 6 different strains/clones (left panel) or among the Kp’s of 204 *L. donovani* strains (right panel). **C.** Spearman correlation matrix used to generate the chord diagram in figure 3B in the main text. Mainly polysomic chromosomes are highlighted in red. Correlations with p-value higher than 0.05 are not shown. **D.** Karyotype Network of the ‘super-mosaic’ population. Color of the nodes indicate the cluster to which each karyotype belongs. Yellow: Cluster A; Red: Cluster B; Green: Cluster C; Blue: Cluster D. Inter-cluster connections were removed. **E.** Comparison of the relative frequency of changes in somy across karyotypes between the 3 groups of chromosomes. ** = p.value < 0.01, *** = p.value <0.001 and **** = p.value <0.0001 (T-test).


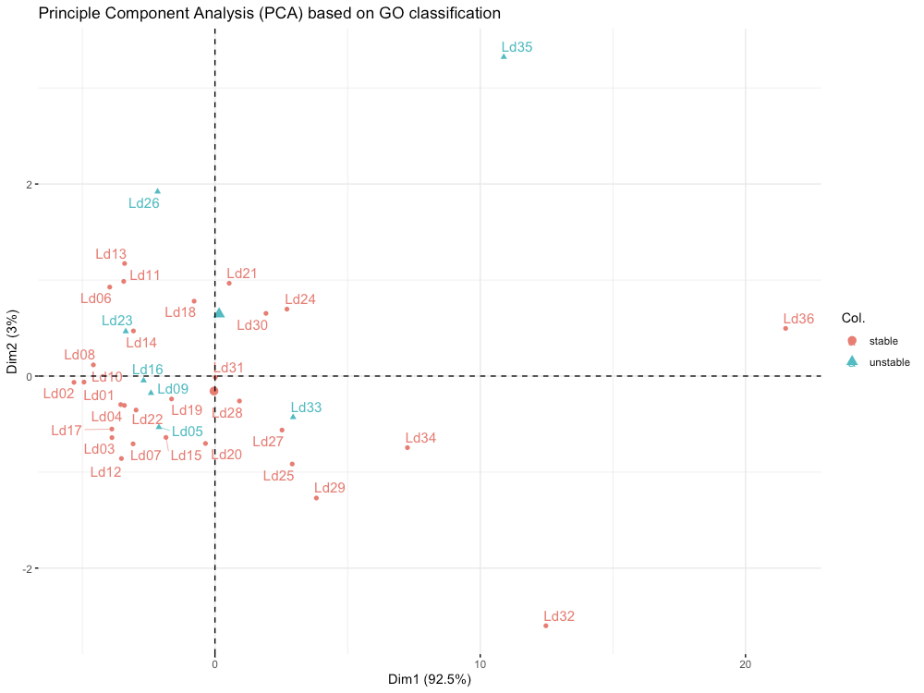


**Supplementary figure 6** – **A.** Principal component analysis (PCA) based on the Gene Ontology (GO) annotation. Based on the GO annotation provided by TriTrypDB, the percentage of each GO category (minimal category size set to 10, maximum category size set to 500), the chromosome by GO category percentage matrix is used as input for the PCA analysis. Chromosomes indicated as “stable” due to their stable disomy are indicated in red, chromosomes which showed frequent changes in ploidy level are indicated in cyan. No obvious clustering of unstable chromosomes is observed based on their GO classification **B.** Heatmap showing the ratio of the genes assigned to a GO class over the total number of genes per GO class (colour code between 0% and 10%), calculated per chromosome. The list of GO classes shown in this heatmap are significantly enriched promastigote-specific GO classes, derived based on the transcriptomics data as available in Dumetz et al. 2017, and are grouped over the three main categories i.e. Biological Process (BP), Cellular Compartment (CC) and Molecular Function (MF). No clear clustering of polysomy-prone chromosomes based on the GO classification was observed.

**A**

**B**


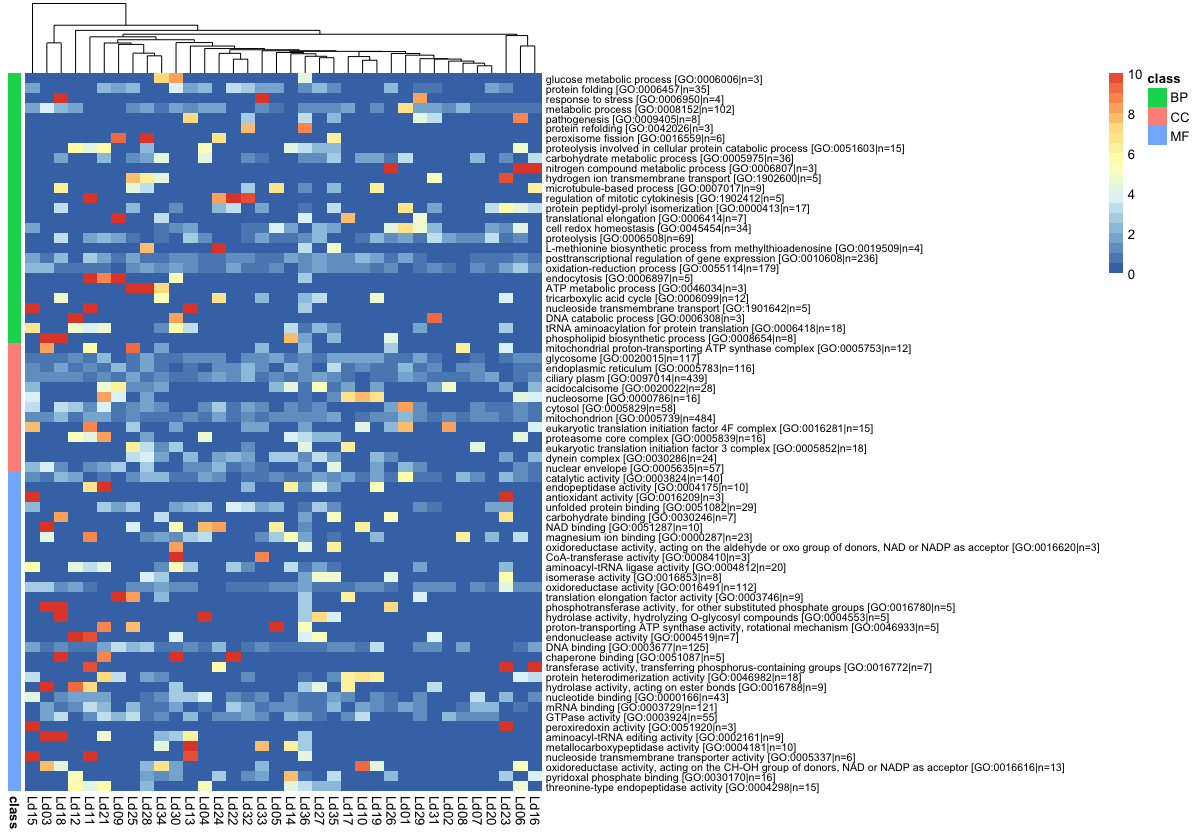

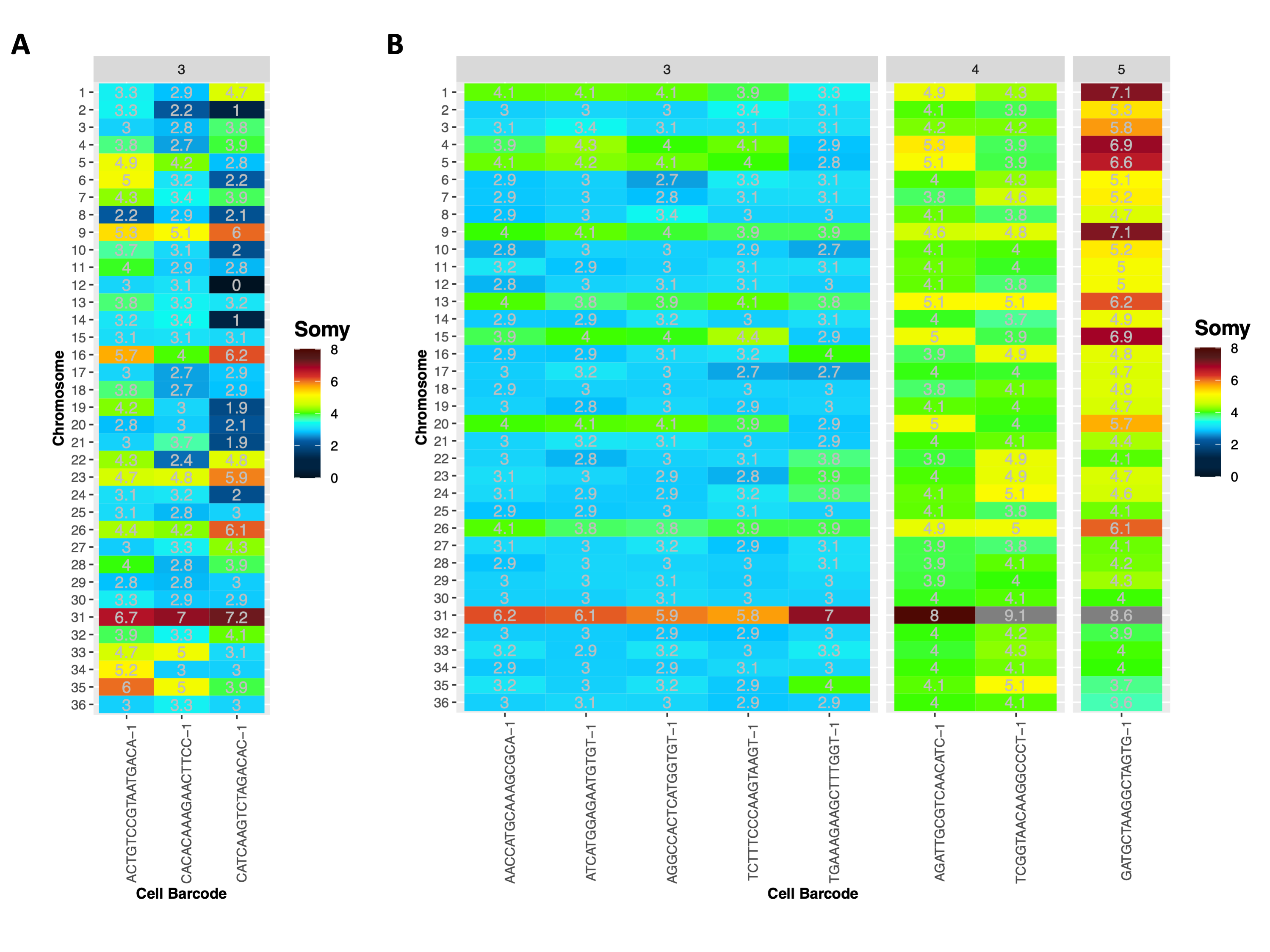


**Supplementary figure 7** - Raw somy values of potentially polyploid cells in BPK282 cl4 (**A**) and BPK081 cl8 (**B**) clones. Plots are separated by the baseline ploidy of the cells (indicated in the top).

**Supplementary Figure 8** – Aneuploidy profile of the cells identified as doublets in the ‘super mosaic’ sample. Doublets displaying karyotypes which are found in other cells are represented in the left panel, while doublets with unique karyotypes are in the center panel. Doublets that were removed from analysis due to high intra-chromosomal variation and therefore did not have their somy values converted to integers are separated in the right panel, displaying their raw somy values instead. The integer somy values (left and center panels) or the raw somy values (right panel) are numerically indicated inside the heat map.


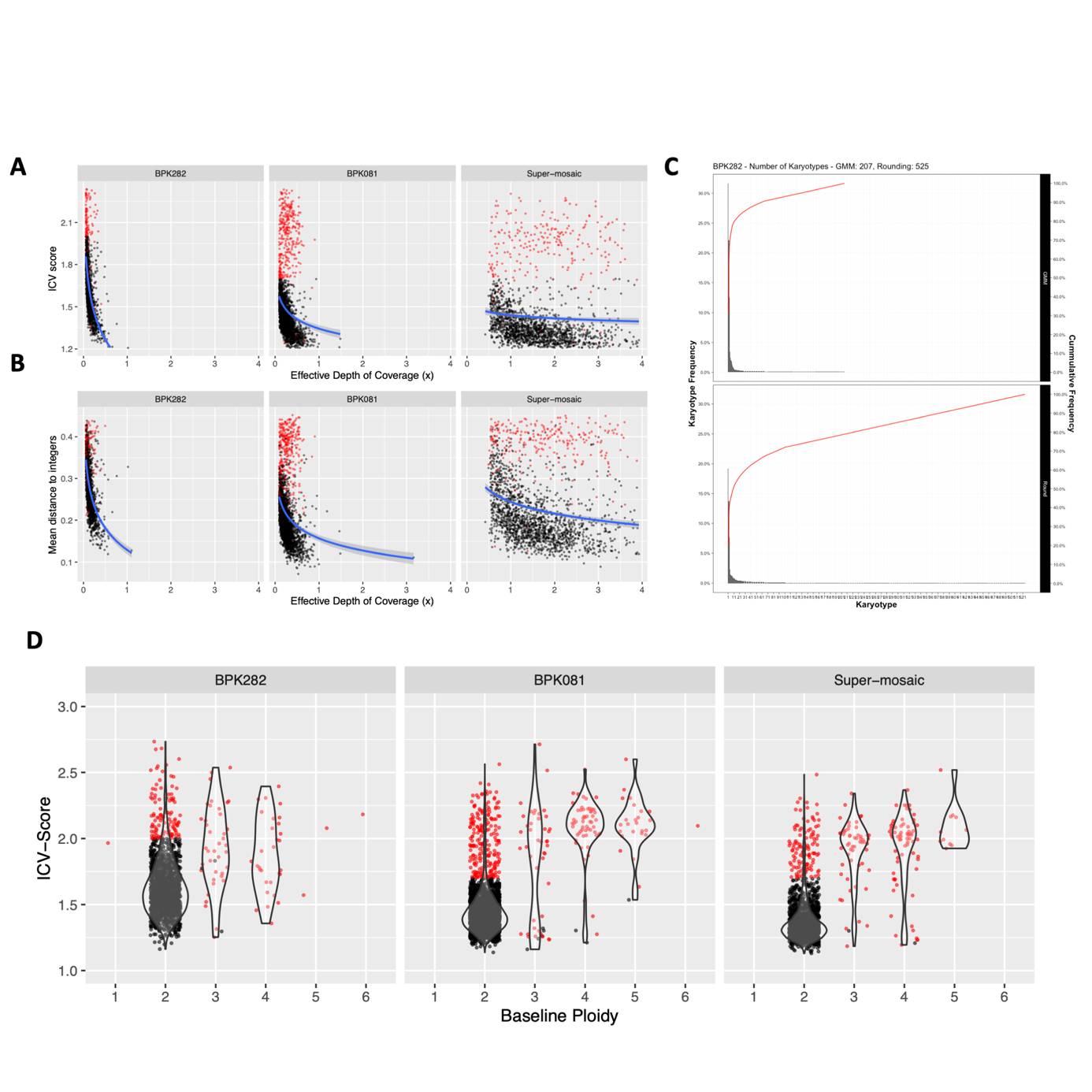


**Supplementary figure 9** – The relationship between the depth of coverage per cell and the cells ICV-score (**A**), mean distance to integers (**B**) and the relationship between the baseline ploidy defined for a cell – which is a direct consequence of the cells scale factor. Red dots represent cells which were removed from karyotype estimation. **C.** Comparison of the number and distribution of karyotypes identified in BPK282 when using the GMMs (top) and when raw somies are simply rounded to their closest integers (bottom). **D.** Relationship between the baseline ploidy defined for a cell and the cells ICV-score.
